# Centromere protein I promotes hepatocellular carcinoma progression by activating PI3K/AKT/mTOR-CDK2 cascade

**DOI:** 10.1080/15384047.2026.2667596

**Published:** 2026-05-12

**Authors:** Demeng Liu, Peihao Yang, Jiyao Wang, Junle Li, Xin Wang, Xiaohui Gu, Chao Liu, Yan Fang

**Affiliations:** a Department of General Surgery, The Second Affiliated Hospital of Zhengzhou University, Zhengzhou, China; b Institute of Clinical Pharmacology, Basic Medical College, Zhengzhou University, Zhengzhou, China; c Department of General Surgery, The Fifth Affiliated Hospital of Zhengzhou University, Zhengzhou, China; d Department of Pharmacy, The Second Affiliated Hospital of Zhengzhou University, Zhengzhou, China

**Keywords:** Centromere protein I, hepatocellular carcinoma, PI3K/AKT/mTOR pathway, cyclin-dependent kinase 2, epithelial-mesenchymal transition, cell cycle

## Abstract

**Background:**

Hepatocellular carcinoma (HCC) is characterized by high recurrence rates and limited targeted therapies. Centromere protein I (CENPI), a core kinetochore component linked to chromosomal instability, is dysregulated in multiple malignancies, yet its role and mechanism in HCC progression remain incompletely elucidated.

**Methods:**

CENPI expression was quantified in paired human HCC tissues and orthotopic rat HCC models via immunohistochemistry (IHC) and western blot (WB). Gain- and loss-of-function assays were performed in HepG2 cells and validated in Hep3B cells to evaluate the effects of CENPI modulation on cell proliferation, migration, invasion, apoptosis, and cell cycle dynamics. Mechanistic analyses included pathway enrichment and WB, with validation via rapamycin in HepG2 cells and LY294002 in Hep3B cells. In vivo tumor growth and signaling alterations were assessed in orthotopic HCC models following CENPI silencing.

**Results:**

CENPI was upregulated in HCC tissues and orthotopic tumors, correlating with poor survival. CENPI depletion suppressed proliferation, migration, invasion, and epithelial-mesenchymal transition (EMT), while enhancing apoptosis and inducing G1 arrest; overexpression was pro-oncogenic. Mechanistically, CENPI activated the PI3K/AKT/mTOR-CDK2 axis, upregulating CDK2, and modulating EMT markers. Rapamycin abrogated CENPI-induced oncogenic signaling in vitro, and CENPI silencing reduced in vivo tumor burden by 65% while suppressing the pathway and EMT.

**Conclusion:**

CENPI may function as an oncogenic regulator in HCC through activation of the PI3K/AKT/mTOR-CDK2 cascade, linking cell cycle progression to EMT-associated invasiveness. These findings provide a preclinical rationale for further evaluating CENPI and its related signaling axis as potential prognostic and therapeutic targets in broader HCC models and clinical cohorts.

## Introduction

HCC remains a major cause of cancer mortality worldwide. Recent global estimates indicate a sustained rise in cancer incidence and death, with primary liver cancer contributing substantially to this burden.^1^^,^^2^ Contemporary clinical guidance has emphasized that HCC management requires risk-adapted surveillance, stage-specific therapy selection, and integration of liver function with tumor burden.^3^ In advanced-stage disease, systemic therapy has evolved rapidly. Immune checkpoint inhibitor (ICI)-based combinations have improved overall survival compared with sorafenib, as demonstrated by the IMbrave 150 regimen (atezolizumab plus bevacizumab) and the HIMALAYA STRIDE regimen (tremelimumab plus durvalumab).^4-6^ Extended follow-up further supports durable benefit in subsets of patients, yet primary resistance, acquired resistance, recurrence, and metastatic dissemination continue to limit long-term disease control.^4^^,^^5^ Accordingly, molecular drivers that connect tumor cell proliferation with invasion-associated plasticity remain actively sought, particularly those that may identify biologically defined subsets for mechanistic study and rational therapeutic testing.

Genomic instability is recognized as a fundamental enabling feature of cancer evolution. Chromosomal instability (CIN) and aneuploidy can generate intratumoral heterogeneity and have been linked to malignant progression and therapeutic adaptation.^6^^,^^7^ Because faithful chromosome segregation depends on the centromere-kinetochore apparatus, deregulation of centromere/kinetochore components has increasingly been implicated in oncogenesis. Overexpressed kinetochore genes have been reported to correlate with copy number variation and proliferative transcriptional programs across multiple cancer types, supporting the concept that altered kinetochore modules may be repeatedly selected during tumor evolution.^7^^,^^8^ Centromere proteins (CENPs) are core constituents of the centromere/kinetochore machinery and are essential for chromosome segregation fidelity. A growing body of work has associated aberrant CENP family expression with aggressive phenotypes and unfavorable outcomes across cancers.^9^

CENPI is a constitutive kinetochore factor required for centromere function. Previous studies have suggested that CENPI is not restricted to a single tumor context but may act as a broader oncogenic and prognostic factor across malignancies. For example, CENPI overexpression has been associated with chromosome instability and poor outcome in estrogen receptor-positive breast cancer.^10^ Recent studies in gastric cancer and breast cancer further support its role in promoting proliferation, migration, and malignant progression.^11^^,^^12^ In addition, pan-cancer analyses have identified CENPI as a potential diagnostic and prognostic biomarker in multiple tumor types. However, tissue-level protein validation and mechanistic characterization in HCC remain insufficient. Accordingly, clarifying whether CENPI is aberrantly expressed in HCC and whether it contributes functionally to malignant phenotypes is an important unresolved question.

One candidate conduit is the phosphatidylinositol 3-kinase/protein kinase B/mechanistic target of rapamycin pathway. Aberrant PI3K/AKT/mTOR activation is widely involved in tumor growth, metabolism, survival, and therapy resistance across malignancies and is commonly implicated in HCC pathobiology.^13^^,^^14^ Beyond its established biological role in tumor growth and survival, the PI3K/AKT/mTOR pathway has remained an active therapeutic target in oncology. Recent reviews have highlighted ongoing clinical development of PI3K-, AKT-, and mTOR-directed agents, as well as biomarker-guided strategies for patient stratification.^13^^,^^15^ In HCC specifically, this pathway continues to be considered clinically relevant, with reviews summarizing ongoing efforts to evaluate pathway inhibition either alone or in rational combinations.^14^ Given its central role in coordinating anabolic signaling, cell-cycle entry, and stress adaptation, this pathway represents a plausible mechanistic axis through which CENPI may promote HCC progression.

In parallel, invasion and dissemination in HCC have been associated with EMT, which is related to cellular plasticity. Consensus guidance has emphasized that EMT should be interpreted and reported using clear operational definitions and multiple orthogonal readouts, rather than as a binary state.^16^ PI3K/AKT/mTOR signaling has been reported to intersect with EMT-associated programs across cancers, making it a biologically plausible axis through which proliferative signaling could couple to invasion-associated behavior.^14^^,^^17^

In this study, we aimed to determine whether CENPI is aberrantly expressed in HCC and to define its functional contribution to malignant phenotypes, with a focus on identifying an experimentally supported signaling relay that connects proliferation, cell cycle progression, and EMT-associated marker remodeling. By integrating analyses of human HCC tissues, orthotopic tumor models, in vitro gain- and loss-of-function experiments, transcriptome-informed pathway interrogation, and pharmacological inhibition, we sought to establish whether CENPI functions as an oncogenic driver in HCC and whether its effects are mediated through activation of the PI3K/AKT/mTOR-CDK2 axis.

## Materials and methods

### Establishment of orthotopic HCC model in SD rats

Orthotopic hepatocellular carcinoma (HCC) models were established in 6-to 8-week-old male Sprague–Dawley (SD) rats weighing 180–220 g using Walker 256 cells. Briefly, 1 × 10⁶ Walker 256 cells suspended in 50 μL Matrigel matrix were injected subcapsularly into the left hepatic lobe of each rat. Tumor growth was monitored weekly by manual palpation. At 4 weeks post-inoculation, rats were humanely euthanized by gradual inhalation of 100% carbon dioxide (CO₂) at a displacement rate of 30% chamber volume per minute, followed by cervical dislocation to confirm death. Tumor tissues and their matched adjacent nontumor liver tissues were then harvested for subsequent WB analysis (*n* = 6 paired samples).

SD rats were purchased from Liaoning Changsheng Biotechnology Co., Ltd. (Benxi, Liaoning, China), with the laboratory animal production license number SCXK (Liaoning) 2025-0001. The license was issued by the Department of Science and Technology of Liaoning Province on June 24, 2025, and is valid until June 23, 2030. Inhalation anesthesia was performed using isoflurane (RWD Life Science, China, catalog number R510-22-10). Rats were induced with 3%–5% isoflurane in an induction chamber, and anesthesia was maintained with 1.5%–2.5% isoflurane via a face mask during surgery. Anesthetic depth was verified by loss of pedal withdrawal and corneal reflexes.

All anesthesia and euthanasia procedures were strictly implemented in accordance with the American Veterinary Medical Association (AVMA) Guidelines for the Euthanasia of Animals (2020 Edition) and the National Institutes of Health (NIH) Guide for the Care and Use of Laboratory Animals (8th edition).

### Cell culture and reagents

Human HCC cell lines HepG2 (catalog no SCSP-510), Hep3B (catalog no SCSP-5045), HCCLM3 (catalog no SCSP-5093), and the immortalized normal human liver cell line THLE-2 (catalog no SCSP-5068) were obtained from the Cell Bank of the Chinese Academy of Sciences (CAS), China. Walker 256 cells (catalog no CL-0377) were obtained from Procell Life Science & Technology Co, Ltd, China. Cells were cultured in DMEM medium (Gibco, China, catalog number C11995500BT) supplemented with 10% fetal bovine serum (CELL-BOX, China, catalog number CF-02S-02) and 1% penicillin-streptomycin (Solarbio, China, catalog number P1400) at 37 °C in 5% CO₂. The human cell lines were STR-authenticated, routinely tested for mycoplasma contamination using the MycoAlert PLUS Kit, and used at passages 3–15.

### siRNA transfection

CENPI-targeting siRNAs and a nontargeting control siRNA (si-NC) were synthesized by Sangon Biotech (Shanghai, China). HepG2 cells were seeded in 6-well plates at 2 × 10^5^ cells/well and transfected at 60%–70% confluence using Lipofectamine 8000 (Beyotime, China, catalog number C0533) in DMEM medium, according to the manufacturer's instructions. The final siRNA concentration was 50 nM. At 48 h after transfection, CENPI protein expression was examined by Western blotting, and knockdown efficiency was calculated relative to the si-NC group based on densitometric quantification normalized to GAPDH. Three candidate siRNAs were initially screened, and the sequence showing the highest knockdown efficiency was selected for subsequent functional experiments. Cells were harvested 48 h post-transfection for downstream assays.

### Plasmid construction and transfection

The full-length human CENPI coding sequence was PCR-amplified from HepG2 cDNA and cloned into the pcDNA3.1(+)-FLAG expression vector (Genecopoeia/Genecfps) between the EcoRI and XhoI restriction sites. The recombinant plasmid was verified by Sanger sequencing. For overexpression experiments, HepG2 cells were seeded in 6-well plates at 1.5 × 10^5^ cells/well and transfected with 2.5 μg plasmid DNA using Lipofectamine 8000 according to the manufacturer's protocol. The empty pcDNA3.1(+)-FLAG vector served as the negative control (oe-NC). At 48 h after transfection, CENPI overexpression efficiency was assessed by Western blotting and densitometric analysis relative to the oe-NC group after normalization to GAPDH. Based on repeated optimization experiments, the transfection efficiency was approximately 70% under these conditions, and cells were collected at 48 h for subsequent assays.

### WB analysis

Total protein was extracted using RIPA lysis buffer (Solarbio, China, catalog number R0010) supplemented with PMSF (Solarbio, China, catalog number IP0280), protease and phosphatase inhibitors (Solarbio, China, catalog number P6730), and protein concentration was determined using a BCA protein assay kit (Glpbio, USA, catalog number GK10009). Equal amounts of protein (30 μg per lane) were separated by 10%–12% SDS-PAGE and transferred onto PVDF membranes. After blocking with Protein Free Fast Blocking Western (Servicebio, China, catalog number G2052) for 30 min at room temperature, membranes were incubated overnight at 4 °C with primary antibodies against CENPI (1:1000, Abcepta, China, catalog number AP9618c), CDK2 (1:1000, SAB, China, catalog number 48670), Cyclin D1 (1:1000, SAB, China, catalog number 48497), PI3K (1:1000, SAB, China, catalog number 41339), phospho-PI3K (1:1000, SAB, China, catalog number 12057), AKT (1:1000, SAB, China, catalog number 21155), phospho-AKT (1:1000, SAB, China, catalog number 11124), mTOR (1:1000, SAB, China, catalog number 41187), phospho-mTOR (1:1000, SAB, China, catalog number 12030), E-cadherin (1:1000, SAB, China, catalog number 48801), N-cadherin (1:1000, SAB, China, catalog number 48779), Vimentin (1:1000, SAB, China, catalog number 48952), and GAPDH (1:5000, SAB, China, catalog number 48358). Membranes were then washed three times with TBST (Solarbio, China, catalog number T1082) and incubated with HRP-conjugated secondary antibodies (1:5000, SAB, China, catalog number L3012 for anti-Rabbit, L3032 for anti-Mouse) for 2 h at 37 °C. Protein bands were visualized using an enhanced chemiluminescence reagent and quantified by ImageJ software. GAPDH was used as the loading control for total protein normalization, and phosphorylated PI3K, AKT, and mTOR were normalized to their corresponding total protein levels. All Western blot experiments were independently repeated at least three times. Detailed information for the antibodies and key reagents used in this study is provided in Supplementary Table S1.

### Immunohistochemistry

Paraffin-embedded sections (4 μm) were deparaffinized, rehydrated, and subjected to antigen retrieval in citrate buffer (pH 6.0) using a pressure cooker for 15 min. Endogenous peroxidase activity was blocked with 3% hydrogen peroxide for 10 min. After blocking, sections were incubated overnight at 4 °C with anti-CENPI primary antibody (1:200, Abcepta, China, catalog number AP9618c), followed by incubation with the corresponding HRP-conjugated secondary antibody (1:1000, SAB, China, catalog number L3012 for anti-Rabbit) at 37 °C for 30 min. Immunoreactivity was visualized using DAB, and sections were counterstained with hematoxylin.

For semi-quantitative evaluation, two independent pathologists blinded to the clinical data scored each slide according to staining intensity and the proportion of positively stained tumor cells. Staining intensity was scored as 0, no staining; 1, weak staining; 2, moderate staining; and 3, strong staining. The proportion of positive tumor cells was scored as 0, <5%; 1, 5%–25%; 2, 26%–50%; 3, 51%–75%; and 4, >75%. The immunoreactive score (IRS) was calculated by multiplying the intensity score by the proportion score, yielding a final score ranging from 0 to 12. In cases of discrepant scoring, the slides were jointly reviewed, and a consensus score was assigned.

### Bioinformatics analysis

CENPI expression differences between normal tissues (GTEx; https://gtexportal.org) and tumor samples (TCGA; https://portal.gdc.cancer.gov) were assessed using R (v4.0.3). Statistical analysis (stats, car) and visualization (ggplot2) employed standard differential expression pipelines (FDR < 0.05, log_2_FC > 1).

### CCK-8 assay

Cell proliferation was assessed using the Cell Counting Kit -8 (Glpbio, USA, catalog number GK10001). Cells were seeded in 96-well plates at 3000 cells/well in 100 μL medium. After treatment, 10 μL of CCK-8 reagent was added to each well and incubated at 37 °C for 2 h. Absorbance at 450 nm was measured using a microplate reader. Experiments were performed in triplicate and repeated three times independently.

### Wound healing migration assay

Cells were seeded in 12-well plates and grown to 90%–95% confluence. A sterile 200 μL pipette tip was used to create a straight scratch. After washing with PBS, cells were incubated in serum-free DMEM for 24 h. Wound images were captured at 0 and 24 h using an inverted microscope equipped with a digital camera. Migration distance was quantified using ImageJ software and calculated as: (initial width-final width)/initial width ×100%. Each experiment was performed in triplicate.

### Transwell invasion assay

Invasion assays used 24-well 8 μm Transwells pre-coated with 50 μL 1:8 Matrigel (Corning, USA, catalog number 3422) (serum-free DMEM). 2 × 10^5^ cells suspended in 200 μL serum-free DMEM were seeded in the upper chamber, while the lower chamber was filled with 600 μL DMEM supplemented with 20% FBS as a chemoattractant. Cells were incubated at 37 °C with 5% CO₂ for 24 h, after which noninvading cells on the upper surface of the membrane were carefully removed with a cotton swab. Invading cells on the lower surface were fixed with 4% paraformaldehyde (PFA) for 15 min and stained with 0.1% crystal violet for 20 min. The number of invaded cells was counted in five random fields under an inverted microscope, and the mean number of invaded cells across these five random fields was calculated for subsequent statistical analysis.

### Flow cytometry analysis

Apoptosis: At 48 h post-transfection, cells were washed with cold PBS (KeyGEN, China, catalog number KGL2206-500), resuspended in 1 × binding buffer and assayed by Annexin V-FITC/PI kit (KeyGEN, China, catalog number KGA1102-50). 1 × 10^5^ cells were incubated with 5 μL each reagent for 15 min in the dark, analyzed on a BD FACSCanto II, and total apoptosis was quantified by FlowJo (v10.8.1).

Cell Cycle: Cells fixed in 70% ethanol at 4 °C overnight were stained with 50 μg/mL RNase A and PI (KeyGEN, China, catalog number KGA9101-20) for 30 min at 37 °C in the dark, then analyzed by flow cytometry, with cell cycle distribution quantified via ModFit LT (v5.0).

### Pharmacological inhibition

Rapamycin (MCE, USA, catalog number HY-10219) was reconstituted in dimethyl sulfoxide (DMSO, Solarbio, China, catalog number D8371) at 10 mM, aliquoted, and stored at −80 °C. For rescue experiments, HepG2 cells overexpressing CENPI were treated with 100 nM rapamycin or vehicle control (0.1% DMSO) at 24 h post-transfection and incubated for an additional 24 h before downstream functional assays. LY294002 (Targetmol, USA, catalog number T2008) was reconstituted in DMSO, aliquoted, and stored at −80 °C. For supplementary rescue experiments, Hep3B cells overexpressing CENPI were treated with 20 μM LY294002 or vehicle control at 24 h post-transfection and incubated for an additional 24 h prior to downstream functional assays.

### Statistical analysis

Quantitative data are presented as mean ± standard deviation (SD), and error bars in all bar graphs and line plots represent SD. All in vitro experiments were independently repeated at least three times unless otherwise specified. Statistical analyses were performed using GraphPad Prism v9.5.0 or R v4.1.0. For comparisons between two groups, a two-tailed Student's *t* test was used for normally distributed data, whereas the Mann–Whitney *U* test was used when distributional assumptions were not satisfied. For paired tumor and adjacent non-tumor tissue comparisons, paired Student's *t* test or the Wilcoxon matched-pairs signed–rank test was applied as appropriate. For comparisons among more than two groups with a single factor, one-way ANOVA followed by Tukey's post hoc multiple-comparisons test was used. For proliferation assays measured across multiple time points (e.g., CCK-8 assays), two-way ANOVA followed by Tukey's multiple-comparisons test was applied, with treatment group and time as factors. Pearson correlation analysis was used to assess the association between gene expression levels. Kaplan–Meier survival curves were compared using the log–rank test. All tests were two-sided, and *p* < 0.05 was considered statistically significant.

## Results

### Upregulated CENPI expression in HCC has diagnostic value and correlates with adverse patient survival

**Figure 1.**
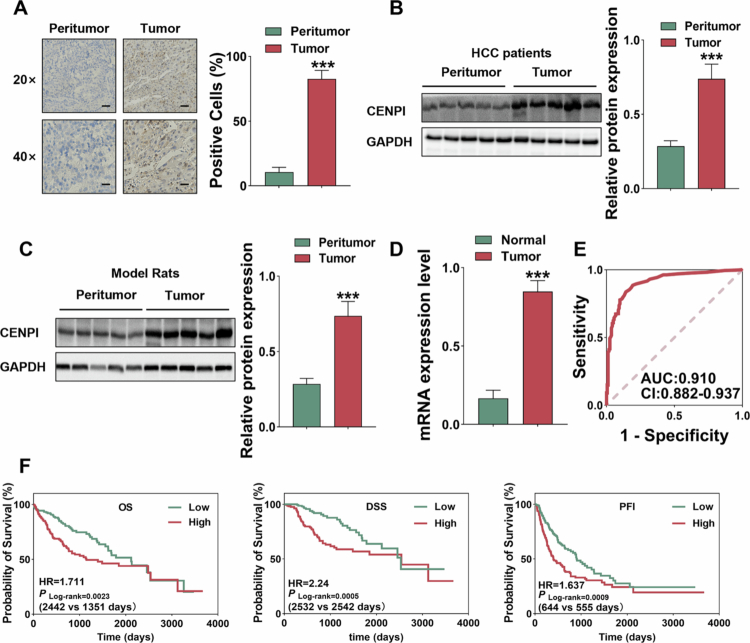
Upregulated CENPI expression in HCC has diagnostic value and correlates with adverse patient survival (A) Representative IHC staining of CENPI in peritumor and tumor tissues from HCC patients; brown staining indicates CENPI-positive signals, scale bars: 20× magnification (100 μm) and 40× magnification (50 μm) (*n* = 10 paired samples), ***p < 0.001. (B) WB analysis of CENPI protein levels in paired peritumor and tumor tissues from HCC patients (*n* = 5 paired samples), ***p < 0.001. (C) WB analysis of CENPI expression in paired peritumor and tumor tissues from the SD rat HCC model (*n* = 5 paired samples), ***p < 0.001. (D) Bar graphs showing CENPI mRNA levels in normal liver tissues (normal, *n* = 110) and HCC tumor tissues (HCC, *n* = 371), the HCC group exhibited significantly higher CENPI mRNA expression than the normal group, ***p < 0.001. (E) ROC curve for the diagnostic value of CENPI mRNA expression in HCC (TCGA-LIHC + GTEx, normal group: *n* = 110; HCC group: *n* = 371); the area under the curve (AUC) was 0.910 (95% confidence interval [CI]: 0.882–0.937). (F) KM survival curves for OS, DSS, and PFI in HCC patients stratified by CENPI mRNA expression (high-expression group: *n* = 187, low-expression group: *n* = 187; grouping based on the median CENPI mRNA level). For quantitative panels, data are presented as mean ± SD, and error bars represent SD.

To investigate the clinical significance of CENPI in HCC, we first examined its expression patterns in clinical samples. IHC staining across the combined main and supplementary cohorts (total *n* = 20 paired HCC and adjacent non-tumor tissues) revealed significantly higher CENPI protein levels in tumor tissues (Figure 1A, S1A). WB analysis across the combined main and supplementary cohorts (total *n* = 10 paired fresh HCC and adjacent nontumor tissues) confirmed that CENPI protein expression was 2.79-fold higher (Figure 1B, S1B). To confirm the high expression of CENPI in HCC, we performed assays using an orthotopic xenograft tumor model of HCC in SD rats, which was independently established by our group. Consistently, in the rat HCC model, CENPI protein levels were also significantly elevated in tumor tissues (Figure 1C), suggesting that CENPI upregulation is evolutionarily conserved across species.

Notably, the mRNA level of CENPI was also validated to be significantly upregulated with a 4.57-fold increase in HCC tissues (*n* = 371) compared with normal liver tissues (*n* = 110). (Figure 1D) from TCGA and GTEx data. ROC curve analysis demonstrated that CENPI mRNA expression had excellent diagnostic performance, with an AUC of 0.910 (Figure 1E). Kaplan–Meier survival analysis revealed that high CENPI expression was associated with significantly worse overall survival, disease-specific survival, and progression-free interval (Figure 1F). Stratified analysis of the TCGA-LIHC cohort further revealed that high CENPI expression was significantly correlated with advanced tumor stage, poor histological differentiation, macrovascular invasion, and elevated AFP levels in HCC patients (Figure S1C), supporting its close association with aggressive clinicopathological phenotypes of HCC. Collectively, these data demonstrate that CENPI possesses robust prognostic potential in HCC and is closely associated with poor clinical outcomes of the disease.

### Proliferation, survival, and invasion phenotypes were reshaped by gain- and loss-of-function perturbation

**Figure 2.**
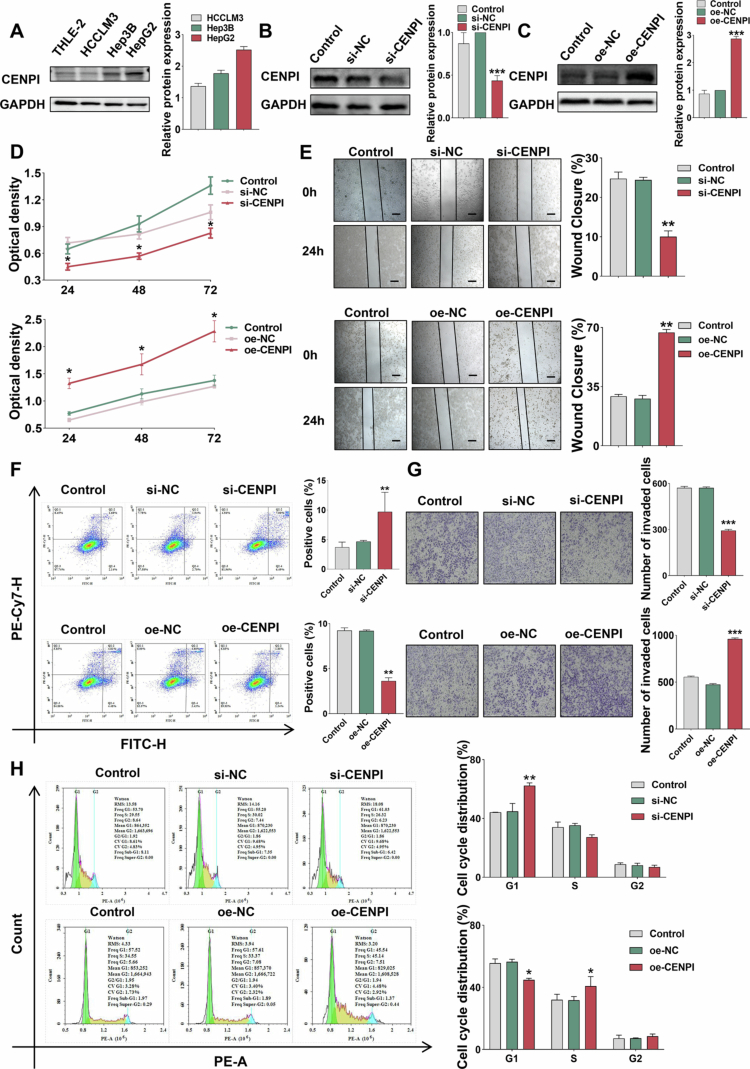
Proliferation, survival, and invasion phenotypes were reshaped by gain- and loss-of-function perturbation. (A) WB analysis of CENPI protein in HCC cell lines (HCCLM3, Hep3B, and HepG2); HepG2 showed the highest CENPI expression (*n* = 3). (B) WB analysis of CENPI protein in si-CENPI-transfected HepG2 cells; si-CENPI decreased CENPI levels vs si-NC, ***p < 0.001 (*n* = 3). (C) WB analysis of CENPI protein in oe-CENPI-transfected HepG2 cells; oe-CENPI increased CENPI levels vs oe-NC, ***p < 0.001 (*n* = 3). (D) CCK-8 assay for HepG2 proliferation; si-CENPI decreased absorbance (weaker proliferation) vs si-NC, while oe-CENPI increased it (stronger proliferation) vs oe-NC, *p < 0.05 (*n* = 3). (E) Wound-healing assay for HepG2 migration; healing status recorded at 0/24 h. si-CENPI decelerated wound closure vs si-NC, while oe-CENPI accelerated it vs oe-NC; scale bar = 100 μm, **p < 0.01 (*n* = 3). (F) Flow cytometry for HepG2 apoptosis; apoptotic rates quantified. si-CENPI increased apoptotic rate vs si-NC, while oe-CENPI decreased it vs oe-NC, **p < 0.01 (*n* = 3). (G) Transwell assay for HepG2 invasion in Matrigel-coated chambers; invaded cells stained and counted. si-CENPI reduced invaded cells vs si-NC, while oe-CENPI increased them vs oe-NC; scale bar = 275 μm, ***p < 0.001 (*n* = 3). (H) Flow cytometry for HepG2 cell cycle; si-CENPI increased the proportion of cells in G1 phase and decreased that in S phase vs si-NC, **p < 0.01, while oe-CENPI showed the opposite effect vs oe-NC, *p < 0.05 (*n* = 3). Data are presented as mean ± SD of three independent experiments; error bars represent SD.

To explore the functional role of CENPI in HCC, we first assessed its expression across three HCC cell lines. WB analysis revealed that HepG2 cells exhibited the highest CENPI expression, followed by Hep3B and HCCLM3 (Figure 2A). Based on this expression pattern, HepG2 cells were used for initial gain- and loss-of-function analyses, and the major phenotypic and mechanistic findings were further validated in Hep3B cells.

For loss-of-function analysis, transfection with siRNA targeting CENPI (si-CENPI) resulted in a 66.41% reduction in CENPI protein levels compared to si-NC controls (Figure 2B). Meanwhile, to eliminate the potential off-target effects of siRNA and verify the generalizability of our findings across different HCC cell lines, we performed functional validation experiments using more than two independent siRNA sequences targeting CENPI in Hep3B cells (Figure 2B, S2A). Conversely, for gain-of-function analysis, overexpression of CENPI (oe-CENPI) was confirmed, showing a significant increase in protein levels compared to oe-NC controls (Figure 2C).

Functional assays demonstrated opposing effects upon CENPI modulation. CCK-8 assays showed that si-CENPI significantly inhibited HepG2 and Hep3B cells' proliferation (Figure 2D, S2B), with the most pronounced effect at 72 h, whereas oe-CENPI significantly enhanced proliferation at the same time point (Figure 2D).

Migration and invasion capabilities were similarly regulated. Wound-healing assays performed in HepG2 and Hep3B cells showed that wound closure was significantly decreased at 24 h after si-CENPI transfection (Figure 2E, S2C), but a significant enhancement in migratory capacity with oe-CENPI (Figure 2E). Flow cytometry analysis of apoptosis revealed that CENPI knockdown potently promoted cellular apoptosis compared to controls in HepG2 and Hep3B cells (Figure 2F, S2D), whereas its overexpression obviously inhibited apoptotic processes (Figure 2F). Furthermore, Transwell invasion assays showed a notable decrease in invaded cell numbers after CENPI knockdown in HepG2 and Hep3B cells (Figure 2G, S2E), in contrast to a distinct increase in invasive potential upon its overexpression (Figure 2G). Cell cycle analysis in HepG2 and Hep3B cells indicated that si-CENPI induced a prominent G1 phase arrest, evidenced by a significant elevation in the proportion of G1-phase cells and a corresponding reduction in S-phase cells (Figure 2H, S2F). Conversely, CENPI overexpression effectively accelerated cell cycle progression, resulting in a marked increase in the proportion of S-phase cells and a concomitant decrease in G1-phase cells. (Figure 2H).

Taken together, these data demonstrate that CENPI knockdown suppresses multiple malignant phenotypes, while its overexpression enhances HCC cell malignancy, supporting a pro-tumorigenic role for CENPI in HCC cells.

### The cell cycle and mTOR programs emerged as dominant signatures, accompanied by CDK2 elevation and EMT marker remodeling

**Figure 3.**
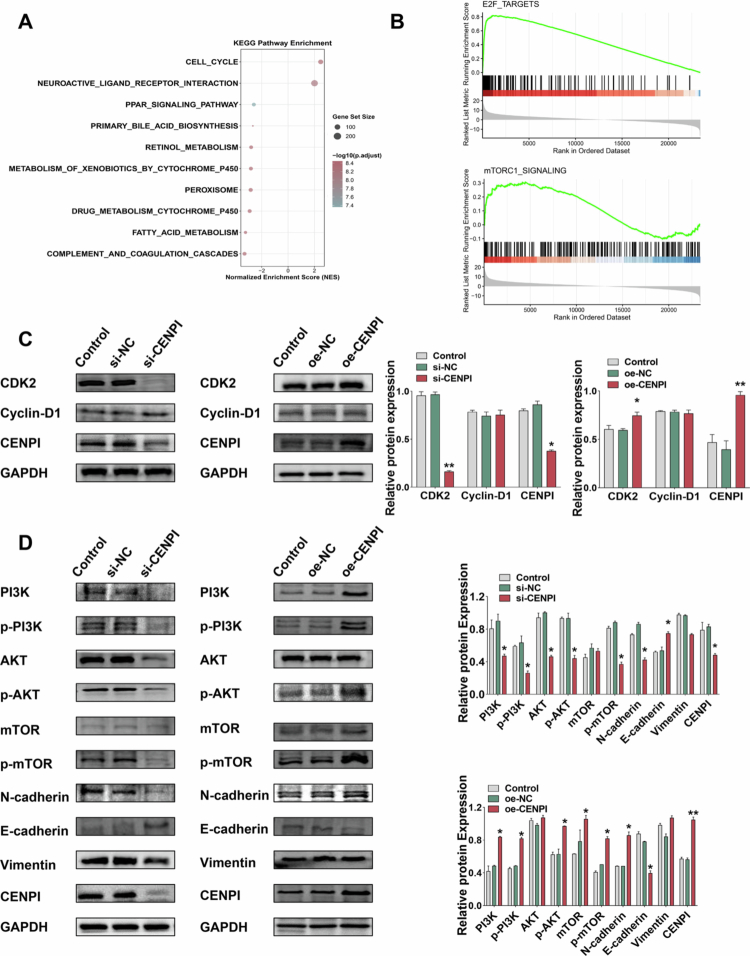
Cell cycle and mTORC1 programs emerged as dominant signatures accompanied by CDK2 elevation and EMT marker remodeling. (A) KEGG pathway enrichment analysis (top ten significantly enriched pathways); the cell cycle pathway was most significant, with significance represented by −log10 (*p* value), based on CENPI-related differentially expressed genes. (B) GSEA analysis of CENPI-related genes; enriched pathways include cell cycle-related (E2F_TARGETS) and mechanism-related (mTORC1_SIGNALING), significance represented by normalized enrichment score (NES). (C) WB analysis of cell cycle-related proteins (CDK2, Cyclin D1) and CENPI in si-CENPI and oe-CENPI-transfected HCC cells; CDK2 showed the most obvious change, **p < 0.01 vs si-NC and *p < 0.05 vs oe-NC (*n* = 3). (D) WB detection of PI3K/AKT/mTOR pathway proteins (PI3K, p-PI3K, AKT, p-AKT, mTOR, p-mTOR), EMT proteins (N-cadherin, E-cadherin, Vimentin), and CENPI in si-CENPI and oe-CENPI-transfected HCC cells, *p < 0.05 and **p < 0.01 (*n* = 3). Quantitative data are presented as mean ± SD of three independent experiments; error bars represent SD.

To elucidate the molecular mechanisms underlying CENPI-mediated HCC progression, we performed KEGG pathway enrichment and GSEA using CENPI-related differentially expressed genes. KEGG analysis identified the cell cycle pathway as the most significantly enriched (Figure 3A). Furthermore, GSEA revealed significant enrichment in E2F_TARGETS (NES = 3.66) and mTORC1_SIGNALING (NES = 1.37) (Figure 3B). Prompted by these findings, Western blot validation showed that CDK2 protein expression was markedly decreased in si-CENPI-transfected cells, whereas it was significantly elevated in oe-CENPI cells. In contrast, Cyclin D1 protein expression remained unaltered in both the si-CENPI and oe-CENPI groups (Figure 3C), indicating a specific regulatory effect of CENPI on CDK2. Consistently, differential expression analysis of the TCGA-LIHC cohort confirmed that CDK2 expression was significantly upregulated in CENPI-high HCC tissues (Figure S3A). Subsequently, Pearson correlation analysis verified a significant positive correlation between CENPI and CDK2 mRNA expression (log2 FPKM) in HCC samples (Figure S3B), which further validated the positive regulatory relationship between CENPI and CDK2 observed in our WB assays.

Furthermore, si-CENPI treatment led to a prominent reduction in both the total expression and phosphorylation levels of key components within the PI3K/AKT/mTOR signaling pathway, with the total mTOR protein level remaining unchanged. Conversely, CENPI overexpression resulted in a significant upregulation of the total expression and phosphorylation of these PI3K/AKT/mTOR pathway proteins, while the total AKT protein level was not affected by CENPI overexpression. Analysis of EMT markers demonstrated that si-CENPI significantly increased E-cadherin expression and concurrently reduced the expression of N-cadherin and Vimentin, which is indicative of suppressed EMT progression. In contrast, CENPI overexpression caused a notable decrease in E-cadherin expression and a marked increase in N-cadherin expression (Figure 3D), thereby promoting EMT. Importantly, similar changes were also observed in Hep3B cells following CENPI knockdown, where reduced CDK2 expression, unchanged Cyclin D1 levels, reversal of EMT-associated marker patterns, and attenuation of PI3K/AKT/mTOR signaling were detected by WB (Figure S3C), indicating that the signaling association was not restricted to HepG2 cells. Collectively, these results support that CENPI promotes malignant phenotypes in HCC in association with activation of the PI3K/AKT/mTOR-CDK2 signaling axis.

### Pharmacologic mTOR blockade attenuated the overexpression-associated phenotype spectrum and reduced CDK2

**Figure 4.**
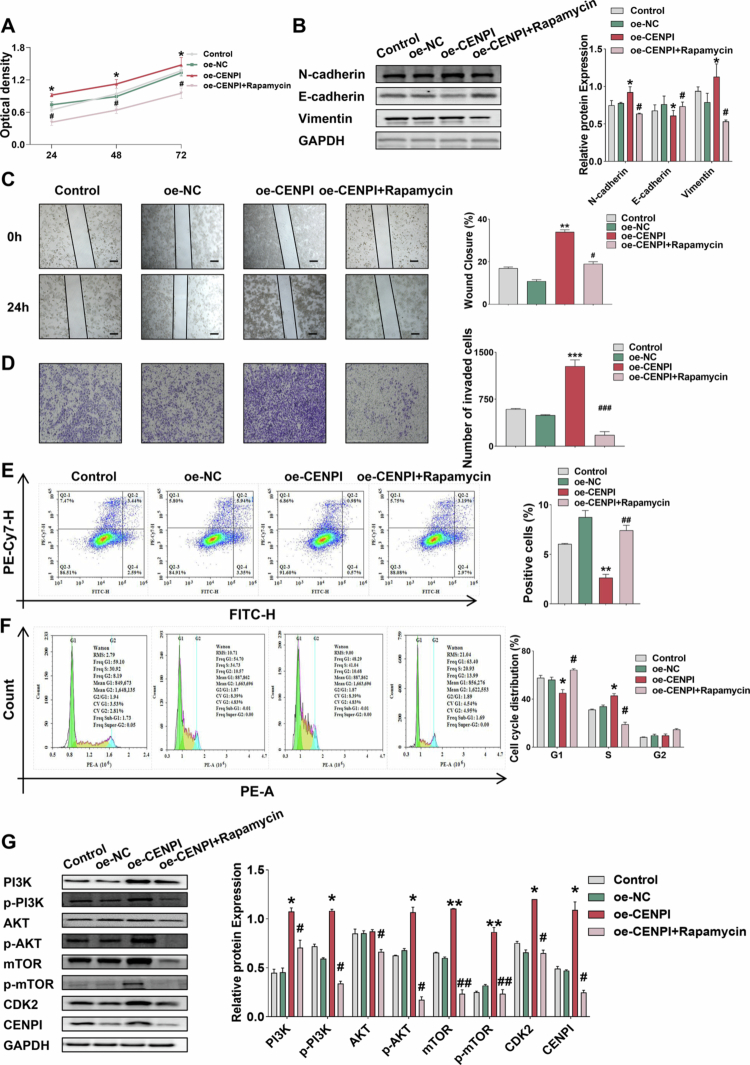
Pharmacologic mTOR blockade attenuated the overexpression-associated phenotype spectrum and reduced CDK2. (A) CCK-8 assay for HepG2 proliferation; oe-CENPI increased absorbance (stronger proliferation) vs oe-NC, while Rapamycin reversed this effect, *p < 0.05 vs oe-NC and ^#^*p* < 0.05 vs oe-CENPI (*n* = 3). (B) WB analysis of E-cadherin, N-cadherin, Vimentin in HepG2. oe-CENPI decreased E-cadherin and increased N-cadherin/Vimentin, with changes mitigated by Rapamycin, *p < 0.05 vs oe-NC and ^#^*p* < 0.05 vs oe-CENPI (*n* = 3). (C) Wound-healing assay for HepG2 migration; healing status recorded at 0/24 h, while Rapamycin reduced migration ability; scale bar = 100 μm, **p < 0.01 vs oe-NC and ^#^*p* < 0.05 vs oe-CENPI (*n* = 3). (D) Transwell assay for HepG2 invasion in Matrigel-coated chambers; invaded cells stained and counted. oe-CENPI increased invaded cells vs oe-NC, with Rapamycin reversing this effect; scale bar = 275 μm, ***p < 0.001 vs oe-NC and ^###^*p* < 0.001 vs oe-CENPI (*n* = 3). (E) Flow cytometry for HepG2 apoptosis; apoptotic rates quantified. oe-CENPI decreased apoptotic rate, while Rapamycin increased it, **p < 0.01 vs oe-NC and ^##^*p* < 0.01 vs oe-CENPI (*n* = 3). (F) Flow cytometry for HepG2 cell cycle; Rapamycin altered cycle (reduced S phase, increased G1 phase) in oe-CENPI-transfected cells, *p < 0.05 vs oe-NC and ^#^*p* < 0.05 vs oe-CENPI (*n* = 3). (G) WB analysis of PI3K/AKT/mTOR pathway proteins (PI3K, p-PI3K, AKT, p-AKT, mTOR, p-mTOR), CDK2, and CENPI in HepG2. oe-CENPI increased PI3K, p-PI3K, p-AKT, mTOR, p-mTOR, CDK2 vs oe-NC with increases reversed by Rapamycin, *p < 0.05; **p < 0.01 vs oe-NC, ^#^*p* < 0.05, and ^##^*p* < 0.01 vs oe-CENPI (*n* = 3). Data are presented as mean ± SD of three independent experiments; error bars represent SD.

To confirm that the PI3K/AKT/mTOR pathway mediates CENPI's oncogenic functions, we performed rescue experiments with Rapamycin (a selective mTOR inhibitor) in HepG2 cells and LY294002 (a PI3K inhibitor) in Hep3B cells. CCK-8 assays showed that Rapamycin significantly reversed CENPI overexpression-induced proliferation, with reduced absorbance at all time points; the most prominent effect was at 72 h (Figure 4A). Similarly, LY294002 also attenuated the proliferation-promoting effect of CENPI overexpression in Hep3B cells (Figure S4A). WB analysis revealed that rapamycin abrogated CENPI-induced EMT, with a notable restoration of E-cadherin expression and a significant reduction in N-cadherin and Vimentin levels, respectively (Figure 4B).

Functional assays further verified that rapamycin abolished the promigratory and proinvasive phenotypes induced by CENPI overexpression. Wound-healing assays showed a marked decrease in migratory capacity at 24 h in rapamycin-treated oe-CENPI cells (Figure 4C), while Transwell invasion assays demonstrated a significant reduction in the invasive potential of these cells (Figure 4D). Flow cytometry analysis revealed a prominent increase in the apoptotic rate of oe-CENPI cells following rapamycin treatment (Figure 4E). Additionally, cell cycle analysis indicated a significant accumulation of cells in the G1 phase and a concomitant marked decrease in the proportion of S-phase cells in the oe-CENPI + rapamycin group (Figure 4F). Consistently, LY294002 treatment in Hep3B cells also reduced migration, increased apoptosis, and partially reversed the cell cycle changes induced by CENPI overexpression (Figure S4B–D).

Mechanistically, WB analysis demonstrated that rapamycin caused a dramatic reduction in CENPI protein expression in oe-CENPI cells, accompanied by a pronounced decrease in the phosphorylation levels of key PI3K/AKT/mTOR signaling components (p-PI3K, p-AKT, and p-mTOR). A significant downregulation was also observed in the total protein levels of PI3K, AKT, and mTOR in these cells (Figure 4G). Similarly, LY294002 attenuated CENPI overexpression-induced activation of the PI3K/AKT/mTOR pathway in Hep3B cells (Figure S4E). Collectively, these results confirm that pharmacologic inhibition of the PI3K/AKT/mTOR axis suppresses the activation of the CENPI-induced PI3K/AKT/mTOR-CDK2 signaling axis and reverses CENPI-mediated oncogenic phenotypes in HCC cells, thus providing additional preclinical support for pathway-focused therapeutic evaluation in CENPI-high HCC.

### Orthotopic growth suppression was accompanied by pathway deactivation and reversal of EMT marker directionality

**Figure 5.**
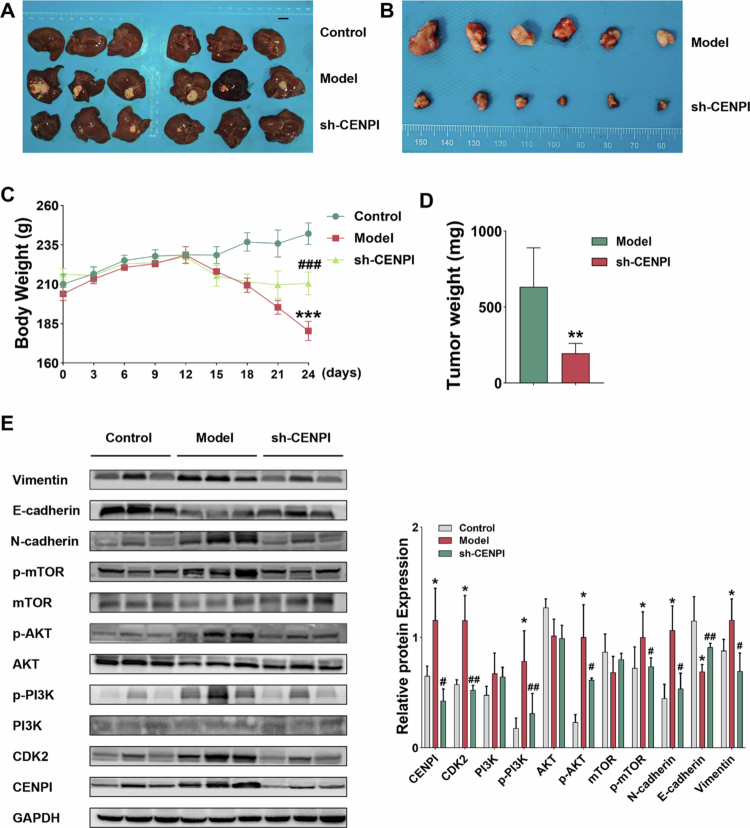
Orthotopic growth suppression was accompanied by pathway deactivation and reversal of EMT marker directionality. (A and B) Quantification of tumor weight in orthotopic xenograft models; shRNA-mediated CENPI silencing reduced tumor weight compared with models; scale bar = 1 cm, (C) Assessment of body weight changes; CENPI silencing attenuated cachexia-driven body weight loss, ***p < 0.001 vs control and ^###^*p* < 0.001 vs model (*n* = 6). (D) Measurement of final tumor mass; CENPI silencing decreased tumor mass vs models without overt hepatotoxicity, **p < 0.01 vs model (*n* = 6). (E) WB analysis of EMT markers (E-cadherin, N-cadherin, and Vimentin) and PI3K/AKT/mTOR-CDK2 pathway proteins in resected tumor tissues. CENPI silencing induced a mesenchymal-to-epithelial reverting signature, with E-cadherin increased and N-cadherin/Vimentin suppressed. Concomitantly, p-PI3K, p-AKT, p-mTOR, and total CDK2 levels were diminished, indicating inactivation of the PI3K/AKT/mTOR-CDK2 relay, *p < 0.05 vs control, ^#^*p* < 0.05, and ^##^*p* < 0.01 vs model (*n* = 3). Data are presented as mean ± SD; error bars represent SD.

Orthotopic xenograft models were established to investigate the in vivo oncogenic functions of CENPI in HCC. Compared with vehicle-treated control rats, shRNA-mediated CENPI silencing significantly reduced tumor weight (Figure 5A, B) and alleviated cachexia-induced body weight loss (Figure 5C), with a marked decrease in final tumor mass observed in the CENPI-silenced group (Figure 5D) and no overt hepatotoxicity detected in these animals. WB analysis of resected tumor tissues revealed a distinct suppression of EMT: E-cadherin expression was markedly upregulated, whereas the expression of N-cadherin and Vimentin was profoundly downregulated. Concomitantly, the PI3K/AKT/mTOR-CDK2 signaling cascade was significantly inactivated, as evidenced by a marked reduction in the phosphorylation levels of PI3K, AKT, and mTOR, along with a significant decrease in total CDK2 protein expression (Figure 5E). These findings corroborated our in vitro signaling data and confirmed that CENPI acts as an essential nodal activator of HCC tumor growth in vivo.

## Discussion

In the present work, CENPI was found to be upregulated at the protein level in HCC tissues and to be associated with unfavorable outcome patterns in cohort analyses. Functional studies showed that proliferative capacity, migration/invasion-associated behavior, apoptosis resistance, and G1/S progression were enhanced by CENPI overexpression and reduced by CENPI knockdown. Mechanistic analyses prioritized cell-cycle and mTOR-related programs, and biochemical validation demonstrated concordant changes in PI3K/AKT/mTOR activation and CDK2 abundance following CENPI modulation. Importantly, rapamycin attenuated both pathway readouts and CENPI-driven phenotypes, supporting mTOR dependence for the measured outputs under the tested conditions. In an orthotopic model, CENPI silencing reduced tumor burden and was accompanied by decreased PI3K/AKT/mTOR signaling, reduced CDK2, and reversal of EMT marker directionality. Together, the data support a model in which CENPI promotes HCC progression through a PI3K/AKT/mTOR-CDK2 signaling relay coupled to EMT-associated marker remodeling in the examined systems. The relatively low basal CENPI expression observed in HCCLM3 cells, despite their highly metastatic phenotype, suggests that intrinsic invasive potential across HCC cell lines is governed by multiple molecular determinants and does not necessarily correlate linearly with basal CENPI abundance.

A central implication is that deregulation of a kinetochore-associated factor can be functionally linked to a canonical growth pathway in HCC. CIN is widely recognized as a hallmark of cancer and is associated with malignant phenotypes and poor clinical outcomes.^6^ Beyond its role in generating karyotypic diversity, CIN and aneuploidy have been proposed as mutation-independent sources of drug resistance and adaptive evolution, emphasizing the translational relevance of chromosomal segregation fidelity.^7^ The observed association between CENPI upregulation and malignant phenotypes aligns with emerging literature describing oncogenic utilization of kinetochore modules, in which overexpression of kinetochore genes correlates with proliferative subnetworks and genomic alterations across cancers.^12^ Prior studies have further shown that CENPI itself is associated with chromosome instability, poor prognosis, and malignant progression in other tumor types, including estrogen receptor-positive breast cancer, gastric cancer, and pan-cancer settings.^10^^,^^11^^,^^18^ In HCC specifically, recent syntheses have summarized accumulating evidence that multiple CENPs are repeatedly deregulated during hepatocarcinogenesis, supporting the view that centromere/kinetochore components represent a nonrandomly selected class of tumor-associated factors.^9^

Mechanistically, the PI3K/AKT/mTOR pathway constitutes a plausible node through which CENPI may exert growth advantages, as this axis integrates growth factor signaling with anabolic metabolism, survival, and cell-cycle entry.^10^ Within HCC, PI3K/AKT/mTOR dysregulation has been reviewed as a recurrent driver of tumor progression and therapeutic vulnerability, and recent literature has further highlighted ongoing clinical development of PI3K-, AKT-, and mTOR-directed agents, as well as biomarker-guided strategies and rational combination approaches for pathway-targeted intervention.^13-15^ In the present study, pathway activation and increased total and phosphorylated levels of PI3K, AKT, and mTOR were observed upon CENPI overexpression, while CENPI knockdown decreased these protein levels and suppressed signaling.

More broadly, our findings can also be interpreted within the context of recent bioinformatics advances in HCC, in which integrative analyses of transcriptomic datasets, protein–protein interaction networks, survival-associated gene modules, and drug–gene interaction resources are increasingly used to prioritize hub genes with potential therapeutic relevance. Recent studies have shown that such approaches can identify highly connected candidate regulators in HCC and place them within clinically relevant signaling frameworks, thereby providing a useful basis for translating gene-level dysregulation into testable therapeutic hypotheses.^19^ In parallel, other novel cell division-related prognostic biomarkers in HCC have also been reported to correlate with tumor immune microenvironment infiltration, suggesting that dysregulated proliferative regulators may influence not only tumor-intrinsic growth programs but also the immune contexture of the disease.^20^ Although the present study was not designed to dissect immune infiltration experimentally, our functional validation of CENPI as an oncogenic regulator supports the broader concept that biologically important hub-like genes in HCC may serve not only as prognostic indicators but also as candidates for mechanistically informed therapeutic stratification and future immunogenomic investigation.

Another key finding is the coordinated remodeling of EMT-associated markers and invasion-associated behavior under CENPI modulation. Here, reciprocal changes in E-cadherin and N-cadherin/vimentin, together with consistent alterations in migration and invasion assays and in vivo marker directionality, support that CENPI promotes EMT-associated marker remodeling with functional invasion correlates in the tested models. Because PI3K/AKT/mTOR signaling has been linked to EMT-associated phenotypes in broader oncology literature, the observed rapamycin sensitivity provides a mechanistic bridge between growth signaling and invasion-associated plasticity in this dataset. This interpretation also supports the possibility that pathway-directed intervention in CENPI-high tumors may influence not only proliferative signaling but also invasion-associated cellular plasticity, although this translational hypothesis requires further validation. In addition, increasing evidence indicates that non-cell-autonomous mechanisms within the HCC microenvironment, particularly exosome-mediated intercellular communication, can regulate chemoresistance, EMT, cancer stemness, autophagic responses, and stromal or immune remodeling.^21^ From this perspective, the CENPI-associated signaling program described here may operate within a broader tumor ecosystem in which exosome-dependent crosstalk contributes to invasive behavior and therapeutic adaptation, although this possibility warrants further investigation beyond the scope of the present study.

These findings were generated in the investigated HCC models and further supported by pharmacological inhibition of the PI3K/AKT/mTOR axis; however, the proximal molecular mechanism linking CENPI to PI3K/AKT/mTOR signaling activation, as well as validation in more diverse preclinical models and large clinical cohorts, remains to be elucidated.

Several key limitations of this study should be acknowledged. First, the use of a limited number of siRNA sequences for CENPI loss-of-function assays carries a potential risk of off-target effects, which may compromise the robustness of our phenotypic findings. Second, our pharmacological rescue experiments using rapamycin and LY294002, while providing supportive evidence for PI3K/AKT/mTOR axis involvement, cannot establish absolute causality in isolation, nor rule out contributions from other pathway mediators. The use of single fixed concentrations of rapamycin and LY294002, without dose-gradient analysis to assess pathway inhibition range and sensitivity, further limits the validation of pathway specificity. Third, we have not confirmed a direct molecular interaction between CENPI and this cascade via assays like Co-IP or ChIP, nor performed genetic rescue experiments (e.g., CDK2 overexpression or pathway reactivation in CENPI-knockdown cells) to fully validate signaling specificity. Finally, our in vivo orthotopic HCC model could be further optimized with additional control groups to enhance experimental rigor.

To address these limitations, future studies will employ multiple independent siRNAs, multinode inhibitors of the PI3K/AKT/mTOR axis with dose-gradient validation, direct molecular interaction assays, and optimized in vivo models. Beyond this cell-autonomous framework, CENPI's potential roles in HCC tumor microenvironment remodeling, immune infiltration, and exosome-mediated intercellular communication also warrant further investigation.

In summary, the results support that CENPI is upregulated in HCC and contributes to malignant phenotypes by engaging a PI3K/AKT/mTOR-CDK2 signaling relay while promoting EMT-associated marker remodeling and invasion-associated behavior in the examined models. These findings provide a preclinical mechanistic rationale for further studies to determine whether CENPI-high tumors represent a biologically meaningful subset for pathway-focused investigation. These findings further suggest that CENPI may serve as a potential biomarker for identifying biologically defined HCC subsets that could benefit from pathway-focused therapeutic strategies in future clinical investigation.

## Conclusion

Our study identifies a preclinical mechanism in HCC whereby CENPI upregulation is associated with malignant progression and activation of the PI3K/AKT/mTOR-CDK2 signaling axis. These findings provide new insight into the molecular context of CENPI overexpression in HCC and support CENPI as a potential oncogenic regulator linking cell cycle progression to EMT-associated invasiveness. In this regard, pharmacologic interference with the PI3K/AKT/mTOR pathway provides a preclinical rationale for further evaluating pathway-focused therapeutic strategies in CENPI-high HCC. A critical next step will be to validate the CENPI-PI3K/AKT/mTOR-CDK2 axis in broader HCC models and larger clinical cohorts, and to define its relevance across additional CENPI-high tumor contexts.

## Supplementary Material

Supplementary Figure 4.tif Supplementary Figure 4.tif

Supplementary Figure legends.docx Supplementary Figure legends.docx

Supplementary Table S1.docx Supplementary Table S1.docx

Supplementary_Figure_3.tif Supplementary_Figure_3.tif

Supplementary_Figure_1.tif Supplementary_Figure_1.tif

Supplementary Figure 2.tif Supplementary Figure 2.tif

## Data Availability

Public datasets used in this study are available at Zenodo: https://doi.org/10.5281/zenodo.19018949. Experimental data supporting the results have been presented in the article. Data used to support the findings of this study are available from the corresponding author upon reasonable request.

## Open scholarship

This article has earned the Center for Open Science badges for Open Data and Open Materials. The data are openly accessible at https://doi.org/10.5281/zenodo.19018949; materials are openly accessible at https://doi.org/10.5281/zenodo.19018949.
