# Supplementary material for: Centromere protein I promotes hepatocellular carcinoma progression by activating PI3K/AKT/mTOR-CDK2 cascade
Source: Cancer Biol Ther. 2026 May 12;27(1):2667596. doi: 10.1080/15384047.2026.2667596 (PMC13174019; doi:10.1080/15384047.2026.2667596)
Supplement: Supplementary Figure legends.docx [file KCBT_A_2667596_SM4537.docx]

**Supplementary Figure legends**

**
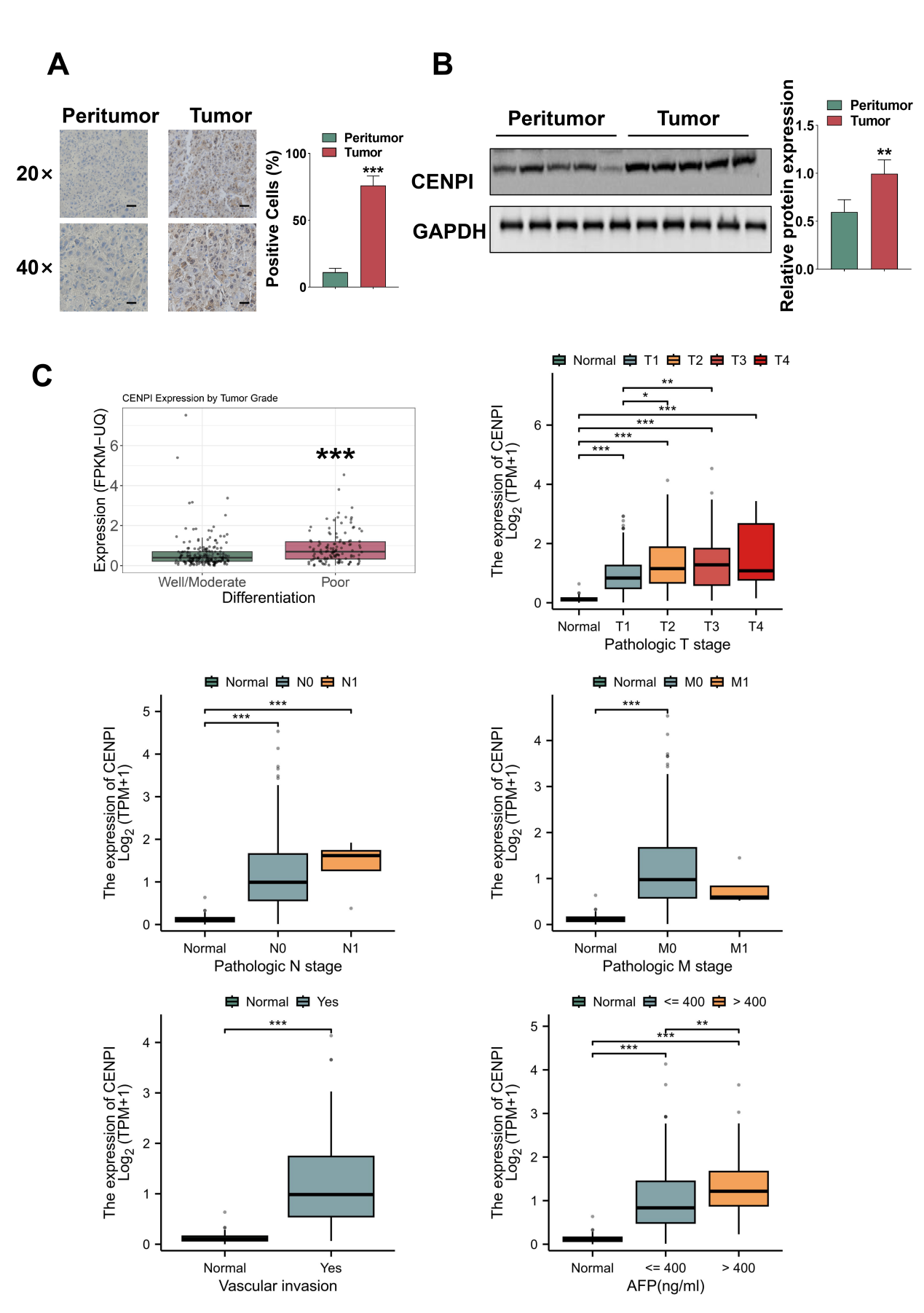
**

**Supplementary Figure 1. Expanded validation of CENPI upregulation in HCC tissues and its correlation with clinicopathological features**

1. Representative IHC staining of CENPI in peritumor and tumor tissues from the expanded HCC patient cohort, with semi-quantitative immunoreactive score (IRS) quantification on the right; brown staining indicates CENPI-positive signals, scale bars: 20× magnification (100 μm) and 40× magnification (50 μm) (n = 10 paired samples), ^***^*P* < 0.001. (B) WB analysis of CENPI protein levels in paired peritumor and tumor tissues from an expanded cohort of HCC patients (n = 5 paired samples), ^***^*P* < 0.001. (C) Box plots showing CENPI mRNA expression levels stratified by tumor stage (n = 371), and histological grade in HCC patients from the TCGA-LIHC and GTEx cohort, with statistical significance between groups indicated (TCGA-LIHC + GTEx, Normal group: n = 110; HCC group: n = 371), ^***^*P* < 0.001. For quantitative panels, data are presented as mean ± SD, and error bars represent SD.

**
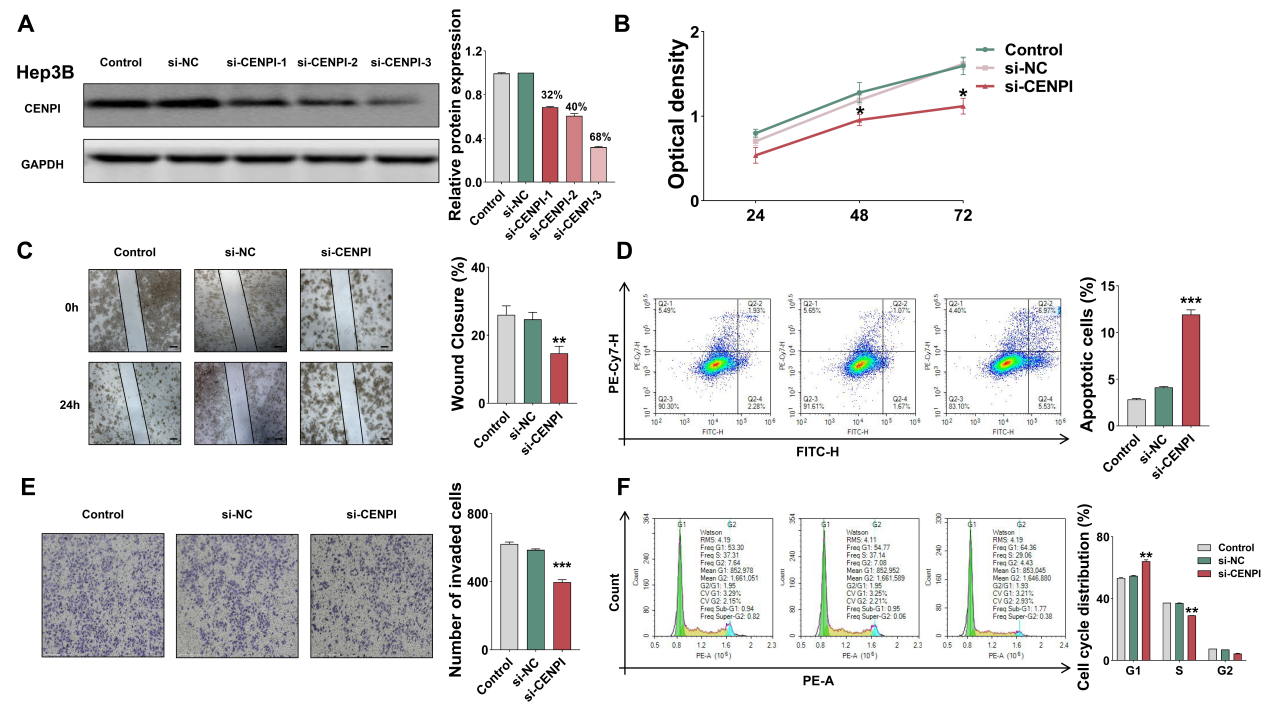
**

**Supplementary Figure 2. Depletion of CENPI impairs malignant behaviors and triggers cell cycle arrest and apoptosis in human hepatocellular carcinoma Hep3B cells**

1. WB analysis of CENPI protein expression in Hep3B cells transfected with si-NC or si-CENPI; si-CENPI transfection reduced CENPI protein levels vs si-NC, (n = 3 independent experiments). (B) CCK-8 assay for Hep3B cell proliferation; si-CENPI transfection significantly decreased the absorbance value (reflecting reduced proliferative capacity) vs si-NC at 72 h, ^*^*P* < 0.05 (n = 3 independent experiments). (C) Wound-healing assay for Hep3B cell migration; healing status recorded at 0/24 h, while si-CENPI transfection reduced migration ability; scale bar = 100 μm, ^**^*P* < 0.01 vs si-NC (n = 3). (D) Flow cytometry analysis of apoptosis in si-NC or si-CENPI-transfected Hep3B cells, with apoptotic rates quantified on the right. si-CENPI transfection significantly increased the apoptotic rate vs si-NC, ^***^*P* < 0.001 (n = 3 independent experiments). (E) Transwell invasion assay for Hep3B cells in Matrigel-coated chambers; invaded cells were stained, counted, and quantified on the right. si-CENPI transfection significantly reduced the number of invaded cells vs si-NC; scale bar = 275 μm, ^***^*P* < 0.001 (n = 3 independent experiments). (F) Flow cytometry analysis of cell cycle distribution in Hep3B cells transfected with si-NC or si-CENPI, with the percentages of G1, S and G2/M phases quantified on the right. si-CENPI transfection significantly increased the proportion of cells in G1 phase and obviously decreased the proportions of cells in S and G2/M phases versus si-NC, ^**^*P* < 0.01 (n = 3 independent experiments). Data are presented as mean ± SD of three independent experiments; error bars represent SD.

**Supplementary Figure 3. Correlation between CENPI and CDK2 expression in HCC tissues and mechanistic validation of associated signaling changes in Hep3B cells**

1. Box plot showing CDK2 mRNA expression (log2 FPKM) in CENPI high-expression and low-expression groups from the TCGA-LIHC cohort (n = 371 HCC samples), *^***^P* < 0.001. (B) Pearson correlation analysis of CENPI and CDK2 mRNA expression levels (log2 FPKM) in HCC tissues from the TCGA-LIHC cohort, with correlation coefficient (R) and *P* value indicated in the plot (n = 371 HCC samples). (C) WB analysis of CENPI, cell-cycle-related proteins (CDK2 and Cyclin D1), EMT-associated markers (E-cadherin, N-cadherin, and Vimentin), and PI3K/AKT/mTOR pathway proteins (PI3K, p-PI3K, AKT, p-AKT, mTOR, and p-mTOR) in Control, si-NC, and si-CENPI-transfected Hep3B cells. CENPI knockdown reduced CDK2 expression, while Cyclin D1 remained largely unchanged, and was accompanied by reversal of EMT-associated marker patterns and attenuation of PI3K/AKT/mTOR signaling. For quantitative panels, data are presented as mean ± SD, and error bars represent SD.


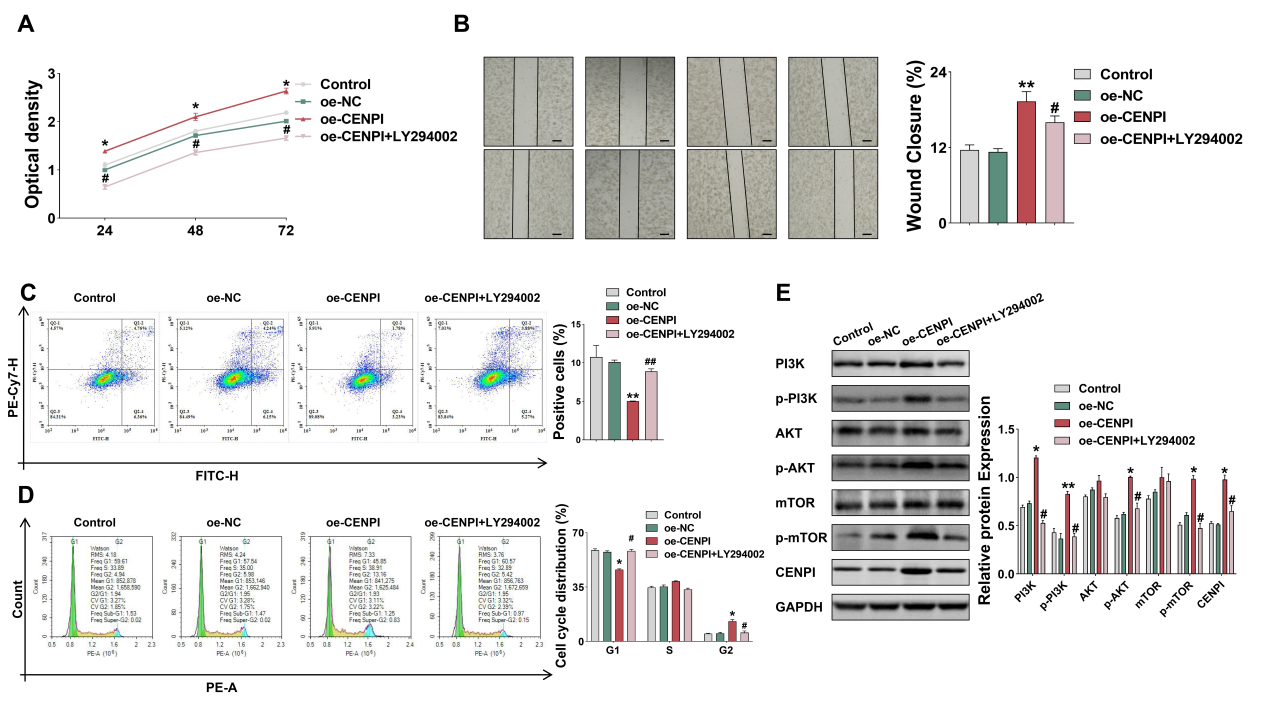


**Supplementary Figure 4. Pharmacologic PI3K blockade attenuated the overexpression-associated phenotype spectrum in Hep3B cells.**

(A) CCK-8 assay for Hep3B proliferation; oe-CENPI increased absorbance (stronger proliferation) vs oe-NC, while LY294002 reversed this effect, ^*^*P* < 0.05 vs oe-NC, ^#^*P* < 0.05 vs oe-CENPI (n = 3). (B) Wound-healing assay for Hep3B migration; healing status recorded at 0/24 h, while LY294002 reduced migration ability; scale bar = 100 μm, ^**^*P* < 0.01 vs oe-NC, ^#^*P* < 0.05 vs oe-CENPI (n = 3). (C) Flow cytometry for Hep3B apoptosis; apoptotic rates quantified. oe-CENPI decreased apoptotic rate, while LY294002 increased it, ^**^*P* < 0.01 vs oe-NC, ^##^*P* < 0.01 vs oe-CENPI (n = 3). (D) Flow cytometry for Hep3B cell cycle; LY294002 altered cycle distribution in oe-CENPI-transfected cells, ^*^*P* < 0.05 vs oe-NC, ^#^*P* < 0.05 vs oe-CENPI (n = 3). (E) WB analysis of PI3K/AKT/mTOR pathway proteins (PI3K, p-PI3K, AKT, p-AKT, mTOR, p-mTOR) and CENPI in Hep3B. oe-CENPI increased p-PI3K, p-AKT and p-mTOR vs oe-NC, with these changes attenuated by LY294002, ^*^*P* < 0.05; ^**^*P* < 0.01 vs oe-NC, ^#^*P* < 0.05 vs oe-CENPI (n = 3). Data are presented as mean ± SD of three independent experiments; error bars represent SD.
