# Supplementary material for: Centromere protein I promotes hepatocellular carcinoma progression by activating PI3K/AKT/mTOR-CDK2 cascade
Source: Cancer Biol Ther. 2026 May 12;27(1):2667596. doi: 10.1080/15384047.2026.2667596 (PMC13174019; doi:10.1080/15384047.2026.2667596)
Supplement: Supplementary Table S1.docx [file KCBT_A_2667596_SM4542.docx]

| **Detailed information for antibodies and key reagents used in this study** | | | |
| --- | --- | --- | --- |
| **Item** | **Company** | **Cat. No.** | **Lot No.** |
| isoflurane | RWD Life Science, China | R510-22-10 | 20250525 |
| DMEM medium | Gibco, China | C11995500BT | 6125383 |
| fetal bovine serum | CELL-BOX, China | CF-02S-02 | 2025022201 |
| penicillin-streptomycin | Solarbio, China | P1400 | 2500120025 |
| RIPA lysis buffer | Solarbio, China | R0010 | 2500050001 |
| PMSF | Solarbio, China | IP0280 | 2500050001 |
| protease and phosphatase inhibitors | Solarbio, China | P6730 | 2500050001 |
| BCA protein assay kit | Glpbio, USA | GK10009 | 106 |
| Protein Free Fast Blocking Western | Servicebio, China | G2052 | 2011G014 |
| CENPI | Abcepta, China | AP9618c | 20200915CYM |
| CDK2 | SAB, China | 48670 | 5106 |
| Cyclin D1 | SAB, China | 48497 | 5814 |
| PI3K | SAB, China | 41339 | 5814 |
| phospho-PI3K | SAB, China | 12057 | 5713 |
| AKT | SAB, China | 21155 | 20211117 |
| phospho-AKT | SAB, China | 11124 | 0489 |
| mTOR | SAB, China | 41187 | 5814 |
| phospho-mTOR | SAB, China | 12030 | 4612 |
| E-cadherin | SAB, China | 48801 | 0248 |
| N-cadherin | SAB, China | 48779 | 0246 |
| Vimentin | SAB, China | 48952 | 0431 |
| GAPDH | SAB, China | 48358 | 0259 |
| TBST | Solarbio, China | T1082 | 2500060009 |
| HRP-conjugated secondary antibodies | SAB, China | L3012 for anti-Rabbit | 6229 |
| HRP-conjugated secondary antibodies | SAB, China | L3032 for anti-Mouse | 6229 |
| Cell Counting Kit -8 | Glpbio, USA | GK10001 | 76 |
| Matrigel | Corning, USA | 3422 | 36525004 |
| PBS | KeyGEN, China | KGL2206-500 | 20250517 |
| Annexin V-FITC/PI kit | KeyGEN, China | KGA1102-50 | 20250312 |
| RNase A and PI | KeyGEN, China | KGA9101-20 | 20250417 |
| Rapamycin | MCE, USA | HY-10219 | 324385 |
| dimethyl sulfoxide | Solarbio, China | D8371 | 34240611006 |
| LY294002 | Targetmol, USA | T2008 | 349945 |
